# Forty years without mental hospitals in Italy

**DOI:** 10.1186/s13033-018-0223-1

**Published:** 2018-07-31

**Authors:** Corrado Barbui, Davide Papola, Benedetto Saraceno

**Affiliations:** 1 WHO Collaborating Centre for Research and Training in Mental Health and Service Evaluation, Department of Neuroscience, Biomedicine and Movement Sciences, Section of Psychiatry, University of Verona, Policlinico GB Rossi, Piazzale Scuro, 10, 37134 Verona, Italy; 2 Lisbon Institute of Global Mental Health, Rua do Instituto Bacteriológico 5, Edifício Amarelo, 1150-190 Lisbon, Portugal

**Keywords:** Italy, Reform law 180, Mental health, Community care

## Abstract

In 1978 Italy implemented Law Number 180, the reform law that blocked all new admissions to public mental hospitals. After 40 years without mental hospitals, we aim at understanding the consequences of the Italian reform in terms of mental health care facility and staff availability. We compared the organization of the Italian mental health system with that of countries belonging to the Group of 7 (G7) major advanced economies. Italy has nearly 8 psychiatrists, 20 nurses, 2 social workers and less than 3 psychologists per 100,000 population, while for example in France there were 22 psychiatrists, in Japan 102 nurses, in the United States 18 social workers, and in Canada and France more than 45 psychologists per 100,000 population. In terms of inpatient facilities, no beds in mental hospitals were available in Italy, while in the other G7 countries mental hospital beds ranged from 8 in the United Kingdom to 204 in Japan per 100 000 population. In Italy there were fewer beds for acute care in general hospitals but more beds in community residential facilities than in the other G7 countries. Service use data showed variability in the provision of mental health care throughout the country. Soon after the implementation of the Italian reform the absolute number of compulsory admissions progressively declined, from more than 20,000 in 1978 to less than 9000 in 2015. Alongside the progressive decline of psychiatric beds imposed by Law 180, the age-adjusted suicide rate remained stable, ranging from 7·1/100,000 population in 1978 to 6·3/100,000 population in 2012. The population of psychiatric patients placed in Italian forensic psychiatric hospitals progressively declined. During the last 40 years without mental hospitals, Italy has seen a progressive consolidation of a community-based system of mental health care. We highlighted, however, reasons for concern, including a decreasing staffing level, a potential use of community residential facilities as long-stay residential services, a still too high variability in service provision across the country, and lack of national data on physical restraints. At a national level, the resources allocated to mental health care are lower in Italy than in other high-income countries.

## Background

A radical change in the organization of mental health care occurred in Italy in 1978 as a consequence of the implementation of the Italian Law Number 180, the reform law that marked the transition from a hospital-based system of care to a model of community mental health care (Box 1) [1–8]. Law 180 blocked all new admissions to public mental hospitals, with immediate effect (i.e. from 1978), as well as readmissions, 2 years later. Consequently, the psychiatric hospital population (78,538 individuals in 1978) dropped by 53% between 1978 and 1987, further declined to 7704 in 1998, and the final dismantling was completed by year 2000 [9, 10].

After 40 years of community mental health care, here we provide an overview of the mental health system in Italy, with emphasis on understanding the consequences of the Italian reform in terms of mental health care facility and staff availability. Using available data taken from both international and national sources (Box 2) [11–15], we compared the organization of the Italian mental health system with that of countries belonging to the Group of 7 (G7) major advanced economies. Additionally, we described trends in compulsory admissions and suicide rates in Italy in the 40 years after the implementation of Law 180.

### Box 1. Summary of the main characteristics of the 1978 Italian psychiatric reform

The main principle of Law 180 is that patients with mental disorders have the right to be treated the same way as patients with other diseases, which means the following:

Acute mental health conditions have to be managed in psychiatric wards located in general hospitals. These wards cannot exceed 15 beds.

Treatments should be provided on a voluntary basis, with compulsory admissions reserved for the following specific circumstances: (1) an emergency intervention is needed; (2) the patient refuses treatment; (3) alternative community treatment is impossible.

Compulsory admissions need to be formally authorized by the Mayor and can only be undertaken in general hospital psychiatric wards.

New community-based services were to be established to provide mental health care to the population of a given catchment area.

Gradual closure of public mental hospitals by blocking all new admissions.

### Box 2. Data source

We used the Organisation for Economic Co-operation and Development (OECD) database to gather information on demographic and economic indicators, psychiatric bed availability and age-standardised suicide rates for Italy and the other G7 countries [11].

The WHO Global Health Observatory [12] and the WHO Mental Health ATLAS-2014 repository [13] were used to extract data on inpatient and outpatient resources for mental health care (both public and private) in Italy and in the other G7 countries. WHO definitions of mental health staff, inpatient and outpatient facilities were used. For inpatient facilities, the following WHO categories were used: mental hospitals (public and private non-profit and for-profit specialized hospital-based facilities that provide inpatient care and long-stay residential services for people with mental disorders), psychiatric wards in general hospitals (public and private non-profit and for-profit psychiatric units usually located within general hospitals that provide inpatient care for the management of acute mental disorders), community residential facilities (public and private non-profit and for-profit non-hospital, community-based mental health facilities that provide overnight residence for people with mental disorders).

From the recently implemented Italian national mental health information system data on the availability and use of mental health facilities (both public and private) were gathered for the year 2015 [14]. The following information was extracted for each Italian region: treated prevalence of any mental disorders (number of individuals with at least one contact with psychiatric services during 2015/10,000 population); treated incidence of any mental disorders (number of individuals with a first ever contact with psychiatric services during 2015/10,000 population); rate of individuals under the care of mental health outpatient facilities (per 10,000 population); rate of individuals under the care of day treatment facilities (per 10,000 population); admissions to community residential facilities (per 10,000 population); admissions to psychiatric wards of general hospitals (per 10,000 population); rate of compulsory admissions (per 10,000 population); proportion of outpatients visits within 30 day after hospital discharge.

As additional source of information, we used the Italian Central Institute of Statistics (ISTAT) data to describe the total number of compulsory admissions and the proportion of all psychiatric admissions that were compulsory from 1978 onwards [15]. Data released from the Commission on psychiatric forensic facilities were used to compute the number of psychiatric patients placed in forensic psychiatric hospitals from 1978 onwards [16].

## Italy in comparison with the other G7 countries

Italy is the fourth most populous European state after Germany, France and the United Kingdom. It hosts a growing proportion of foreign population, which is approaching 10%, as in Germany (Table 1). In 2014, the number of healthy life years at birth was estimated at 83 years, similar to Japan and higher than the other G7 countries. The unemployment rate in 2016 was close to 12%, with a gross domestic product much lower than the other G7 countries. In 2011, the proportion of government expenditures on mental health was half than Germany or France (Table 1).

**Table 1:** Demographic and economic indicators for Italy and the other countries belonging to the Group of 7 (G7) major advanced economies (OECD data)

	Canada	France	Germany	Italy	Japan	UK	USA	Year
Population (million persons)	35.54	64.06	80.89	60.44	127.51	63.65	318.85	2014
Foreign population (% of population)	NA	NA	9.29	8.11	1.62	7.70	6.96	2013
Healthy life expectancy at birth (years)	NA	82.40	81.20	83.20	83.70	81.40	78.80	2014
Unemployment rate (% of labour force)	6.99	10.05	4.10	11.68	3.11	4.80	4.86	2016
Gross domestic product (total, US dollars/capita)	44,025	41,489	48,839	38,146	41,534	42,651	57,325	2016
Government expenditures on mental health (% of total expenditure on health)	7.20	12.91	11.00	5.00	4.94	NA	NA	2011

*OECD* Organisation for Economic Co-operation and Development

*NA* not available

Italy, in comparison with the other G7 countries, has fewer human resources for mental health care (Table 2). According to WHO ATLAS-2014, there were nearly 8 psychiatrists, 20 nurses, 2 social workers and less than 3 psychologists per 100,000 population, while for example in France there were 22 psychiatrists, in Japan 102 nurses, in the United States 18 social workers, and in Canada and France more than 45 psychologists per 100,000 population (Table 2).

**Table 2:** Staff availability and resources for mental health care in Italy and in the other G7 countries

	Canada	France	Germany	Italy	Japan	UK	USA
Staff^a^							
Psychiatrists working in mental health sector (per 100,000)	12.61	22.35	15.23	7.83	10.1	14.63	12.40
Nurses working in mental health sector (per 100 000)	65.0	86.21	56.06	19.28	102.55	67.35	3.07
Social workers working in mental health sector (per 100 000)	NA	3.83	NA	1.93	6.06	1.99	17.93
Psychologists working in mental health sector (per 100 000)	46.56	47.9	NA	2.58	3.99	12.83	29.03
Inpatient facilities^b^							
Beds for mental health in general hospitals (per 100 000)	NA	22.72	41.08	10.95	73.12	50.63	14.36
Beds in community residential facilities (per 100 000)	NA	NA	NA	46.41	16.23	2.28	22.29
Beds in mental hospitals (per 100 000)	31.38	71.81	47.62	0	204.4	7.99	19.44
Outpatient facilities^b^							
Mental health outpatient facilities (per 100,000)	NA	5.75	30.32	1.43	2.31	4.94	1.95
Day treatment facilities (per 100,000)	NA	3.50	0.61	1.34	1.05	2.88	NA

*NA* not available

^a^From WHO Global Health Observatory (GHO)

^b^From WHO ATLAS

In terms of inpatient facilities, no beds in public mental hospitals were available in Italy, as required by Law 180, while in the other G7 countries mental hospital beds showed high variability, ranging from 8 in the United Kingdom to 204 in Japan per 100,000 population. In Italy there were fewer beds for acute care in general hospitals than in the other G7 countries, with Japan having more than 70 beds in general hospitals and Italy around 10/100,000 population. However, In Italy the rate of beds in community residential facilities was higher than in other countries where this information was available (Table 2).

## Trends in public health indicators

**Fig. 1:**
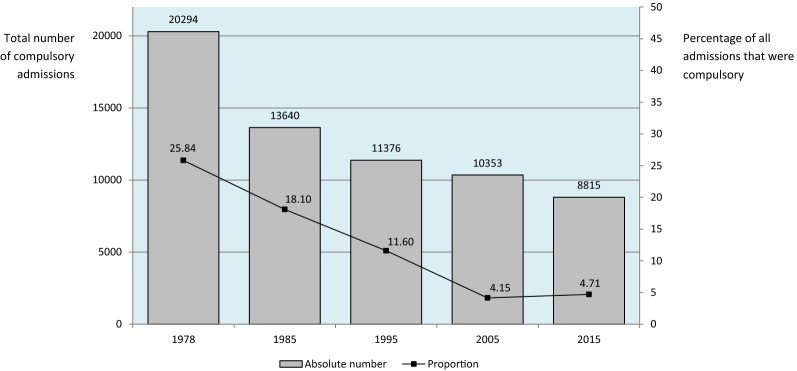
Compulsory psychiatric admissions in Italy, 1978–2015 (ISTAT data)

Soon after the implementation of the Italian reform the absolute number of compulsory admissions progressively declined, from more than 20,000 in 1978 to less than 9000 in 2015. Similarly, the proportion of psychiatric admissions that were compulsory progressively declined from 1978 to 2005, and remained stable thereafter, accounting for less than 5% of all psychiatric admissions (Fig. 1).

**Fig. 2:**
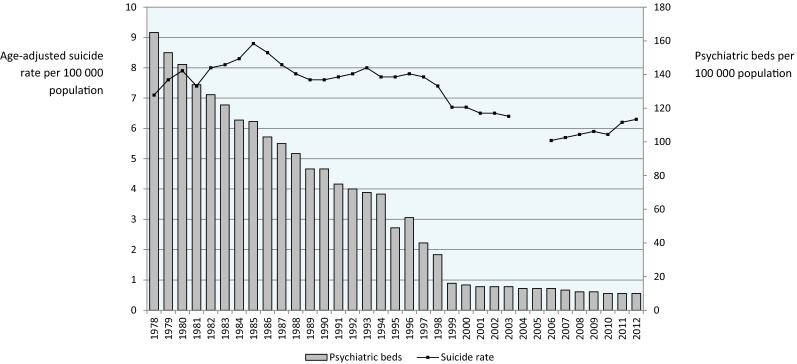
Availability of acute-care psychiatric beds in Italy and age-standardised suicide rates from 1978 to 2012 (OECD data, 2004 and 2005 are missing)

**Fig. 3:**
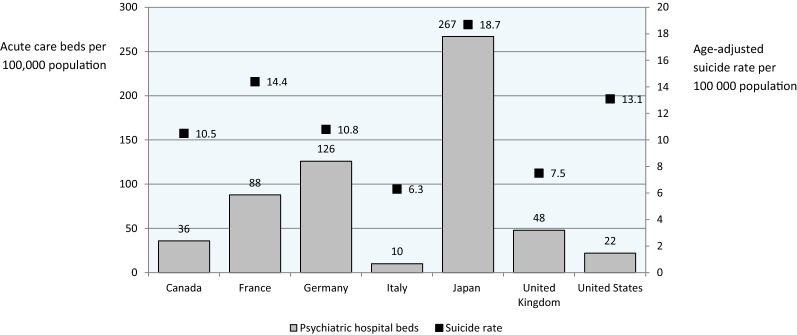
Availability of acute-care psychiatric beds and age-standardised suicide rates in the G7 countries (OECD data)

Figure 2 describes the age-adjusted suicide rate in Italy from 1978 onwards, alongside the progressive decline of psychiatric beds imposed by Law 180. In 1978 there were 7.1 suicides per 100,000 population, while in 2012 there were 6.3 suicides per 100,000 population, with the highest rate in 1985 (8.8 suicides per 100,000) and the lowest in 2006 (5.6/100,000). Lack of a clear relationship between psychiatric bed availability and suicides was also suggested by Fig. 3, where psychiatric beds for the G7 countries are reported alongside the national rate of suicides. In Japan the rate of suicide was the highest among the G7 countries, despite more than 250 psychiatric beds per 100,000 population, while in the United States there were high suicide rates with relatively few psychiatric beds. The United Kingdom showed a situation similar to Italy, with few beds and relatively low suicide rates.

Unfortunately, there is little epidemiological data on the population of psychiatric patients placed in forensic psychiatric hospitals from 1978 onwards. In 1980, the population comprised 1424 people, in 1987 there were 977 people and in 2012 there were 1264 people [16]. In 2016, after the phasing out of forensic psychiatric hospitals, there were 541 individuals placed in newly developed residential facilities providing intensive mental health care to socially dangerous individuals with mental disorders [16]. Additionally, there were 201 individuals with mental disorders placed in psychiatric units in prison, yielding an overall number of 742 people for the year 2016 [16].

## Service use data for mental health care in Italian regions

In Table 3 service use data for mental health care in Italy is presented for the year 2015. Substantial variability in the provision of mental health care can be observed throughout the country. For example, the treated prevalence of mental disorders, a proxy indicator of the coverage capacity of community psychiatric services, ranged from 205 individuals per 10,000 population in Emilia Romagna (north of Italy) to 108 in Basilicata (south). Similar differences were observed for the treated incidence of mental disorders, although a north to south gradient was not observed, being higher in Liguria and Friuli (north of Italy) and lower in Lombardy (north), Tuscany (north), Umbria (centre), Marche (centre) and Basilicata (south). On average, in Italy there were 150 individuals per 10,000 population under the care of mental health outpatient facilities, with wide regional differences, and 6 individuals per 10,000 population under the care of day treatment facilities (Table 3).

**Table 3:** Treated prevalence and treated incidence of mental disorders, and service use data for mental health care in Italian regions, year 2015 (Italian national mental health information system data)

Italian region (north to south)	Rate per 10,000 population							Percentage
	Treated prevalence of mental disorders	Treated incidence of mental disorders (first-ever cases)	Individuals under the care of mental health outpatient facilities	Individuals under the care of day treatment facilities	Admissions to community residential facilities	Admissions to psychiatric wards	Compulsory admissions	Outpatient visits within 30 days after hospital discharge
Piemonte	163.68	67.64	164.13	8.79	5.34	24.20	1.37	52.1
Valle D’Aosta	NA	NA	NA	NA	NA	30.17	2.99	NA
Lombardia	176.65	46.52	172.54	6.70	5.71	23.78	0.96	55.3
Bolzano	NA	NA	NA	NA	NA	40.19	0.22	NA
Trento	165.96	57.54	177.27	4.77	3.70	19.26	0.95	86.7
Veneto	143.40	67.99	142.29	13.98	4.56	28.69	0.98	34.9
Friuli Venezia Giulia	116.52	120.20	113.86	10.45	2.95	6.34	0.43	69.9
Liguria	175.33	131.32	176.26	6.66	10.35	37.46	1.19	41.7
Emilia Romagna	205.82	79.38	206.25	5.54	14.27	26.78	2.64	56.9
Toscana	110.49	39.01	110.66	3.07	3.62	23.23	1.16	49.3
Umbria	164.89	48.03	184.12	3.74	9.79	10.48	1.94	30.1
Marche	158.94	44.05	168.84	4.35	8.84	24.25	5.68	49.7
Lazio	138.60	76.39	131.45	5.59	10.13	17.31	1.46	NA
Abruzzo	142.41	66.83	141.82	4.37	3.88	24.50	1.49	32.1
Molise	165.10	71.77	167.24	2.06	5.05	22.00	1.61	63.6
Campania	139.39	54.05	153.06	3.47	1.56	9.06	1.90	57.8
Puglia	167.58	79.69	159.83	4.27	6.25	17.35	2.07	47.3
Basilicata	107.63	47.98	135.73	2.53	5.94	18.75	0.72	NA
Calabria	161.34	105.85	222.09	0.30	0.37	16.38	2.10	50.0
Sicilia	186.33	90.27	196.19	3.70	4.99	28.40	3.08	42.5
Sardegna	NA	NA	NA	NA	NA	19.48	2.33	NA
Italy	159.40	68.13	153.87	5.91	6.10	21.87	1.73	49.4

*NA* not available

In terms of bed use, there were slightly more than 20 admissions to general hospital beds per 10,000 population, with substantial variability in terms of proportion of patients with an outpatient visit within 30 days after discharge, ranging from nearly 90% in Trento (north) to less than 35% in Veneto (north), 32% in Abruzzo (centre) and 30% in Umbria (centre). The rate of compulsory admissions was 1.73/10,000 population, ranging from 5.68 in Marche (centre) to 0.22 in Bolzano (north) and 0.43 in Friuli (north). On average, there were 6 admissions to community residential facilities per 10,000 population, with substantial variability. Interestingly, the average length of stay in these facilities was higher than 750 days, ranging from 30 days in Campania (south) to 2 269 days (more than 6 years) in Veneto (north).

## Data-based considerations on the Italian experience

It has often been emphasised the closing of mental hospitals as the main objective of the Italian reform, while its first and main aim is that individuals with mental disorders are treated the same way as individuals with other diseases. Implementing this principle has determined a shift in the role and focus of psychiatry, from custody and coercion to treatment and care. All the practical changes to the Italian mental health system have been a consequence of this paradigm shift: the total dismantling of old asylums, the development of psychiatric wards in general hospitals and the implementation of a community-based system of mental health care.

### Compulsory admissions and suicides

A hard indicator of the shift from custody to care is a progressive decline in compulsory admissions, both in terms of absolute numbers and in terms of proportion of psychiatric admissions that were involuntary. In other countries different trends have been observed. In the United Kingdom, for example, the number of uses of the Mental Health Act has been rising, with the highest ever year-on-year rise (10%) to 58,400 detentions in 2014/15 [17]. More than half of admissions to psychiatric hospitals in England are now involuntary, the highest rate recorded since the 1983 Mental Health Act, with wide local variations [18].

A decreasing availability of psychiatric beds has been suggested as one explanation for the rise in compulsory admissions [19]. On similar grounds, in the United States a decreasing availability of psychiatric beds has been suggested as one explanation for the rise in suicide rates [20–23]. The natural experiment offered by the Italian reform would suggest that a direct and linear relationship between psychiatric bed availability and these public health indicators should not be expected. Despite a dramatic decrease in acute-care hospital beds, compulsory admissions decreased and suicide rates remained stable. Data from other G7 countries would reinforce this point, as there are countries with high rates of both beds and suicides, countries with low rates of beds and suicides, and countries with diverging rates. Of course we acknowledge that a wide variety of social, economic, health, mental health and context variables may significantly affect such indicators, and therefore no causal inference can be derived from these descriptive data. However, for the same reasons we argue that increasing the number of psychiatric beds may hardly be considered an evidence-based public health measure to decrease the rates of suicides and the rates of involuntary admissions.

Data available on individuals placed in forensic facilities from 1978 onwards suggests that the phasing out of mental hospitals has not determined an increase of this population, which has declined. Unfortunately, no data are available on the true prevalence of mental disorders in people placed in Italian prisons. A study conducted in one prison found a prevalence of 19.3% of one or more diagnostic and statistical manual of mental disorders, fourth edition, axis I current mental disorders (excluding substance misuse) [24], which seems in line with international estimates [25].

### Mental health workers

Fewer human resources were available in Italy than in other high-income countries. WHO ATLAS showed that the median number of mental health workers per 100,000 population vary from below 1/100,000 population in low-income countries to over 50 in high-income countries [26]. In Italy there were 33 workers per 100,000, which is below the median of 43.5/100,000 population in Europe and below the median of 52.3/100,000 population in high-income countries. The global median is 9/100,000 population, or less than one mental health worker for every 10,000 people [26]. Although it may be argued that the Italian experience suggests that human resources are not as important as system organisation, it is nevertheless true that staff availability is associated with the capacity of providing mental health care which, in turn, affects the coverage for severe mental disorders, which is one of the main targets mentioned by the WHO action plan [27]. Related to this, Italy has the lowest gross domestic product among the G7 countries, with the lowest proportion of government expenditures on mental health. Looking ahead, this may represent a key challenge for the sustainability of the Italian mental health care system, and for the quality of health care provided by mental health services.

### Community residential facilities

In Italy we recorded more beds in community residential facilities as compared with other high-income countries. These are non-hospital, community-based facilities that provide overnight residence for people with mental disorders. Usually these facilities serve individuals with relatively stable mental disorders who require rehabilitation interventions. In Italy both public and private non-profit and for-profit facilities are available. A challenging issue is that a length of stay exceeding 2 years on average, and reaching 6 years in some Italian regions, may suggest that these facilities, rather than focusing on rehabilitation, provide inpatient care and long-stay residential services. This was also suggested by the PROGRES survey, which showed that patients in residential facilities were mostly males, with low education, and with a disability pension in the majority of cases Almost half of the sample surveyed was totally inactive, not even assisting with their facility’s daily activities. Extremely low resident turnover emerged as one of the most relevant problems [28–30]. Looking ahead, we argue that the mission and operational definition of residential facilities should be reconsidered, perhaps recognising that for many long-term, disabled patients, these facilities currently represent ‘‘homes for life’’ rather than rehabilitation sites. In this perspective, we recognise some ambiguity in their role, being focus on rehabilitation and care but also on some degree of protection, with a risk of gently switching back to custody as main mission.

### Variability in service provision

In terms of regional differences, we highlighted a marked variation in service provision for different areas of the country, especially between the more wealthy areas of northern and central Italy and the poorer regions of the south. It was particularly worrying to note a marked variation in the proportion of discharged patients seen within a month, which is an indicator of continuity of care between hospital and the community, an aspect that is usually considered quite strong in the Italian mental health care system. Not only wide differences were observed in different areas of the country, but the average percentage of 49% is well below the average for European countries and for high-income countries, which is 81 and 76%, respectively [13]. Looking ahead, we argue that continuity of mental health care should receive more attention by policy makers and team leaders who have planning and clinical responsibilities, taking advantage of the recently implemented Italian national mental health information system that may play a key role in monitoring this indicator and in providing data to check if poor continuity of care is associated with other facility-related variables, for example the mental health staffing level [31].

### Limitations

The description of the Italian reform presented here has several limitations. A first problem is that national statistics describing health systems may have some imprecisions that cannot be quantified. However, WHO and OECD data are based on operational definitions to decrease ambiguity and to guide towards a common interpretation. WHO ATLAS, for example, has a glossary of terms to precisely characterise facilities, workers, and all the service use data that were collected [13]. A second issue is that national statistics do not capture the type and quality of care provided by Italian mental health facilities. However, at the end of the 1990s, two consecutive nationwide projects gathered an unprecedented amount of data about residential care and acute inpatient care [28–30, 32]. On the whole, the data collected highlighted several critical issues, such as a large regional variability in the availability of residential and acute inpatient beds, a delay between symptom onset and first contact with psychiatric services, and a substantial proportion of patients that seem not to receive fully adequate care [28, 29]. Other studies conducted on large, representative numbers of patients in treatment showed that the quality of mental health care may often be of limited quality [33–35].

## Concluding remarks

Overall, during the last 40 years without mental hospitals, Italy has seen a progressive consolidation of a community-based system of mental health care. The Italian experience would suggest that the number of psychiatric beds may not represent a key factor for public health indicators such as rates of suicides, involuntary admissions, and people placed in forensic facilities. We highlighted, however, reasons for concern, including a decreasing staffing level, a potential use of community residential facilities as long-stay residential services, and lack of community alternatives to acute inpatient admissions. Action is therefore required to reverse these trends. At a national level, the resources allocated to mental health care are lower in Italy than in other high-income countries. Consequently, apart from notable exceptions, the organization of services has remained very similar to that implemented 40 years ago. This does not consider the fact that the Italian society has been profoundly changing and the needs of special populations, for example the elderly and adolescents, as well as the needs of new populations, such as economic migrants, asylum seekers and refugees [36], are not receiving enough consideration in current service planning and delivery [37]. Additionally, very few evidence-based specific interventions and treatment modalities, such as early intervention teams for first-episode psychosis, for example, have been implemented. Italy needs to improve what is called ‘translational epidemiology’ in psychiatry [38].

Policy makers and clinical team leaders, with the involvement of a variety of stakeholders and the wider society, should be able to generate a new and innovative vision for the future of mental health care, motivating all the actors involved to work together, as a team, towards new achievements, aiming for continuous improvement and continuous reinforcement of treatment and care as main mission.
